# Molecular Approach to Cutaneous Squamous Cell Carcinoma: From Pathways to Therapy

**DOI:** 10.3390/ijms21041211

**Published:** 2020-02-12

**Authors:** Elisabetta Palazzo, Maria I. Morasso, Carlo Pincelli

**Affiliations:** 1 Laboratory of Cutaneous Biology, Department of Surgical, Medical, Dental and Morphological Sciences, University of Modena and Reggio Emilia, 41100 Modena, Italy; carlo.pincelli@unimore.it; 2 Laboratory of Skin Biology, National Institute of Arthritis and Musculoskeletal and Skin Diseases, National Institutes of Health, Bethesda, MD 20892, USA; morassom@mail.nih.gov

Cutaneous squamous cell carcinoma (cSCC) represents the second most frequent skin cancer, recently showing a rapid increase in incidence worldwide, with around >1 million cases/year in the United States and 2500 deaths [1]. cSCC can develop either on healthy tissue, or on a pre-existing actinic keratosis (AK) or on a burn scar. In particular, the number of people with AK is increasing rapidly and the AK affects more than 60% of the elderly population. Therefore, the increase in average life in the era of industrialization means that the cSCC represents a major public health problem, with a diagnosis that remains differential and requires biopsy.

So far, cSCC healthcare costs stands in fifth place after prostate, lung, colon, and breast cancers. If detected at an early stage, cSCC can be surgically treated, but it might be disfiguring and costly. However, if left untreated, especially for aggressive cSCC variants, cSCC can rapidly grow and often metastasize. Therefore, the identification of molecular biomarkers or altered gene networks as novel models for dissecting the complexity of cSCC progression, will give a key through which the development of more effective and accessible therapies could be possible. The Special Issue “Molecular Aspects of Cutaneous Squamous Cell Carcinoma” contains contributions by several established investigators in the field, which focus on different aspects of the biology and pathology of cSCC, in both human and animal models. 

In this issue, Sarasamma and co-workers [2] highlight how animal models have key roles in the identification of the pathways involved in the pathobiology of cancers and are fundamental for the development of novel therapeutic tools. Specifically, these authors emphasize that in recent years, a number of important results on skin cancers have been obtained by the use of fish models (teleost, zebrafish, platy fish, and medaka), where skin cancers showed a parallel development to the disease in humans. Moreover, the last part of the review reports the recent molecular technologies applied on skin cancer models in zebrafish, which is an emerging tool for cancer research because of, among other factors, a higher number of animal for each experiment, lower time-course for metastasis evaluation, and low costs [3]. 

Ventura and coworkers provide an in-depth analysis of the telomere and telomerase pathway in cSCC pathogenesis, as compared to basal cell carcinoma and melanoma [4]. Little is known on this topic [5,6,7], but, giving the potential role of telomere biology as a prognostic tool or therapeutic target, the attention on telomere length, TERT promoter mutations or the level of telomerase activity (usually increased in cSCC tumors) and the risk of cSCC, needs further investigation.

Another key player during skin carcinogenesis is p38 [8]. Kiss and colleagues had previously reported that mice with germline deletion of p38δ gene are significantly protected from skin carcinogenesis, exhibiting a marked resistance to the development of skin papilloma after the DMBA/TPA chemical protocol [9]. In the work presented in this issue [10], they report conditional deletion of p38δ in keratinocytes and myeloid cells, showing that the tissue-specific deletion is able to determine the susceptibility to chemical carcinogenesis. Keratinocyte-specific ablation did not influence the latency, incidence, or multiplicity of the skin tumors, but increase the tumor volume in females and reduce malignant progression in males. On the other hand, myeloid-specific ablation inhibited skin carcinogenesis in male mice but not in females. 

A collaborative review by Matthew Bottomley, Jason Thomson, Catherine Harwood, and Irene Leigh discuss the role of the immune system in cSCC [11]. They present the concept of “immunoediting” that describes the process through which the elimination of cancer cells leads to positive selection for those cells that have lost the expression of immunogenic tumor-specific antigens, which in turn can proliferate and develop a more favorable tumor niche. The analysis of immunosuppressed vs. immunocompetent individuals as well as premalignant vs. malignant lesions, with novel computational tools, can dissect the dynamic interactions between the tumors and their microenvironment, including immune cells. 

Among the well-established mechanisms controlling gene expression regulation, microRNA (miRNAs or miRs) are able to perform their control function at the post-transcriptional level [12,13,14]. These small RNA molecules have been implicated in a wide variety of biological processes, such as differentiation, proliferation, survival, and apoptosis, as well as immune modulation, inflammation, metabolic control, and development [15]. In cancer, it is possible to distinguish between oncogenic and oncosuppressor miRNAs depending on the specific pathway where they execute their function. Natalia Garcia-Sancha and collaborators discuss the most recent reports centered on the involvement of miRNAs in the development and prognosis of cSCC [16]. 

cSCC tumors display a complex cellular heterogeneity, with some cells maintaining the expression of differentiation markers, and some other cells being only partially differentiated or undifferentiated. These features may depend on the cells of origin in that particular cSCC. It has been hypothesized that tumors originating from stem cells can develop just because of “few genetic hits”, and cells, even though mutated, still maintain their function until the clonal expansion carrying the favored oncogenic drivers occurs [17]. It is possible to identify a “cancer initiating cell” that develops into cancer stem cell [18], which in turn, gives rise to a population characterized by the expression of specific molecules. The growth of these cells may be influenced by several factors, including surface receptors, miRNAs, or immune-related proteins. In the present issue, Goldie and collaborators have reviewed the latest findings in the oncogenic events in a specific cell population within the cSCC, that could be considered as a novel therapeutic target [19]. 

As stated above, the identification of specific pathways involved in cSCC development and progression has been possible because of the use of animal models and a series of cell lines, which keep some of the tumor features in vitro. Hassan and collaborators, isolated keratinocytes from cSCC tumor and metastasis, obtaining SCC cell lines that underwent genotyping and profiling by the analysis of short tandem repeats. Moreover, these cell lines were further characterized by morphology, growth, aggressiveness, and xenografting ability in vivo. The results showed that it is possible to select a specific cell line according to the aim of the study, and that this panel of SCC cells will be an invaluable tool for in vitro and in vivo research [20]. 

A novel approach to study cSCC tumors is the use of 3D culture such as spheroids. Using this type of cultures, Palazzo and co-workers [21] analyzed the neurotrophin receptor CD271 in SCC cells. This work demonstrates that the expression of this receptor correlates with SCC cell adhesion in tumor spheroids. Moreover, the authors demonstrate that, in healthy epidermis, CD271 is expressed in proliferative progenitor cells and could regulate the expression of the transcription factor DLX3, which in turn, determines p53-dependent regulation of epidermal differentiation and modulation of skin carcinogenesis [22]. Although the role of neurotrophins and their receptors in cSCC is still to be determined, a novel CD271-DLX3 axis in keratinocytes has been proposed as a potential pathway to prevent skin carcinogenesis. 

In general, the role of complement system in the context of cancer is two-folds. While it works against tumor toxicity or stimulates the immune response against tumor, it has also been implicated in cancer progression through different mechanisms [23,24]. Since chronic inflammation is a risk factor for cSCC progression and the complement system is part of the inflammatory cSCC microenvironment, Riihila and co-workers report about the function of each complement component and specific inhibitors as potential new options for treatment [25].

Finally, Moses and collaborators describe the molecular mechanisms through which the dysregulation of Δp63α can contribute to squamous cancer development [26]. The authors analyzed findings related to squamous cancers and epidermal keratinocyte biology as well as other neoplasms which share the overexpression of p63 as a main and distinctive element. They summarize the known transcriptional regulatory mechanisms of p63 (direct promoter binding, interactions with enhancers and chromatin remodeling complex, modulation by non-coding RNA) in the mouse and cellular models utilized up to date. This allowed for a comprehensive summary of the biological processes, mainly dysregulated in cancers, where p63 has important roles (proliferation, differentiation, apoptosis, stem cell maintenance, senescence, cytokine release, adhesion, microenvironmental influences).

In summary, this special issue discusses previous and current strategies, and lines of thought that correlate to cSCC prognosis, development, and progression. 
